# Supplementary material for: Newborns with Favourable Outcomes after Perinatal Asphyxia Have Upregulated Glucose Metabolism-Related Proteins in Plasma
Source: Biomolecules. 2023 Sep 30;13(10):1471. doi: 10.3390/biom13101471 (PMC10604898; doi:10.3390/biom13101471)

Patient samples from different groups were loaded randomly into the wells (NOTE: blot was cut into strips based on the molecular weight of the protein of interest using the protein ladder to allow for multiple staining of different antibodies)

Samples used as representative images cited in the manuscript are highlighted with a red asterisk.

Blot 1 GAPDH

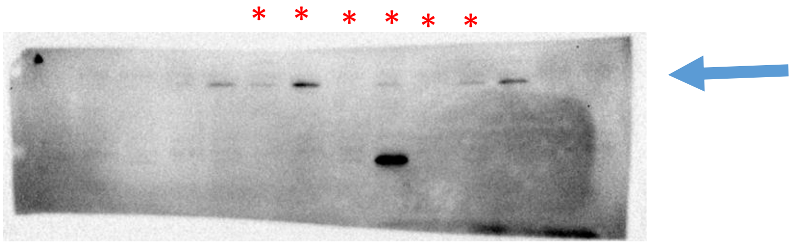

Blot 2 GAPDH

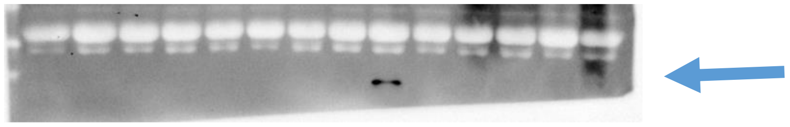

Blot 1 beta-actin

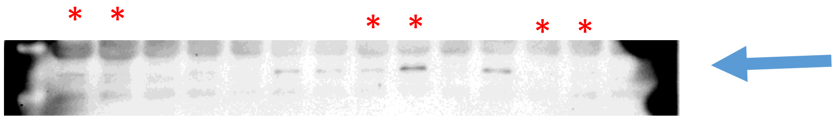

Blot 2 beta-actin

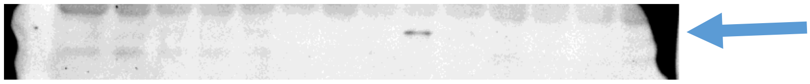

(NOTE: blot was cut into strips based on the molecular weight of the protein of interest from protein ladder to allow for multiple staining of different antibodies)

Blot 1 lactotransferrin

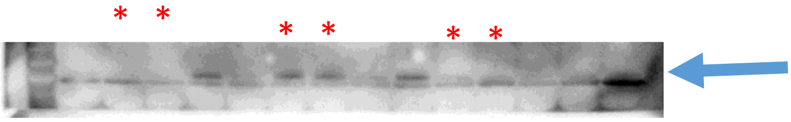

Blot 2 lactotransferrin

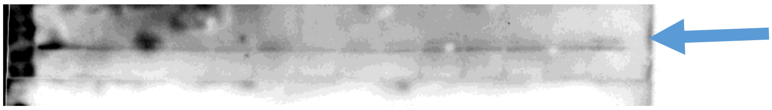

Blot 1 PGK1

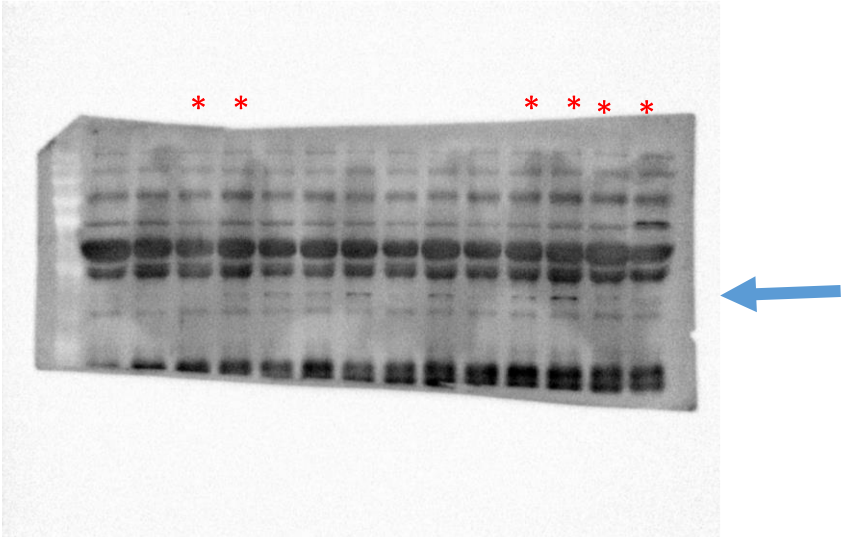

Blot 2 PGK1

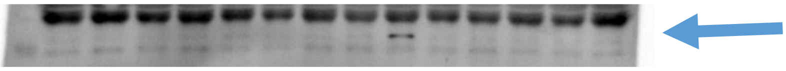

Supplement: Supplementary file 1 [file biomolecules-13-01471-s001.zip › biomolecules-2547161-supplementary.pdf]
